# A Case of Andexanet Alfa Induced Heparin Resistance in Emergent Aortic Surgery: Successful Anticoagulation Management Using Antithrombin Administration

**DOI:** 10.7759/cureus.50856

**Published:** 2023-12-20

**Authors:** Kazuyoshi Takagi, Tomofumi Fukuda, Kosuke Saku, Eiki Tayama

**Affiliations:** 1 Division of Cardiovascular Surgery, Department of Surgery, Kurume University, Kurume, JPN

**Keywords:** act, antithrombin iii, aortic dissection management, heparin resistance, andexanet alfa

## Abstract

Andexanet alfa (AnAl) is utilized for the urgent reversal of direct oral anticoagulants (DOACs) in cases of severe bleeding. While the guidelines from the Society of Thoracic Surgeons recommend AnAl for urgent cardiac surgery in patients treated with DOACs, concerns persist regarding the potential of AnAl to induce heparin resistance. This report details the case of an 85-year-old woman diagnosed with acute type A aortic dissection, who received AnAl due to prior edoxaban use. During the emergent aortic surgery, she exhibited heparin resistance following the administration of unfractionated heparin (UFH). The administration of antithrombin III (ATIII) significantly influenced activated clotting times, facilitating successful surgery while maintaining adequate anticoagulation. This case underscores the importance of cautious management of AnAl-induced heparin resistance during critical surgeries, emphasizing the role of ATIII supplementation for effective anticoagulation.

## Introduction

Andexanet alfa (AnAl), a recombinant coagulation factor Xa decoy protein (Ondexxya, Portola Pharmaceuticals, San Francisco, USA), is approved for emergency reversal of anti-Xa-targeted direct oral anticoagulants (DOACs) in life-threatening or uncontrollable bleeding. However, its specific role in emergent cardiac surgery remains uncertain due to its approval via the accelerated approval program, limiting the scope and enrolled patient population [1,2]. The 2021 Society of Thoracic Surgeons guidelines recommend AnAl for DOAC-taking patients requiring urgent cardiac surgery [3]. However, concerns exist among anesthesiologists regarding AnAl-induced heparin resistance [4]. Additionally, AnAl-induced heparin resistance has solely been documented in case reports, and as of present, the precise incidence as well as the optimal diagnostic and treatment approaches remain undetermined [1,5-10]. We present a case of AnAl-induced heparin resistance managed with antithrombin during emergent aortic surgery.

## Case presentation

**Figure 1:**
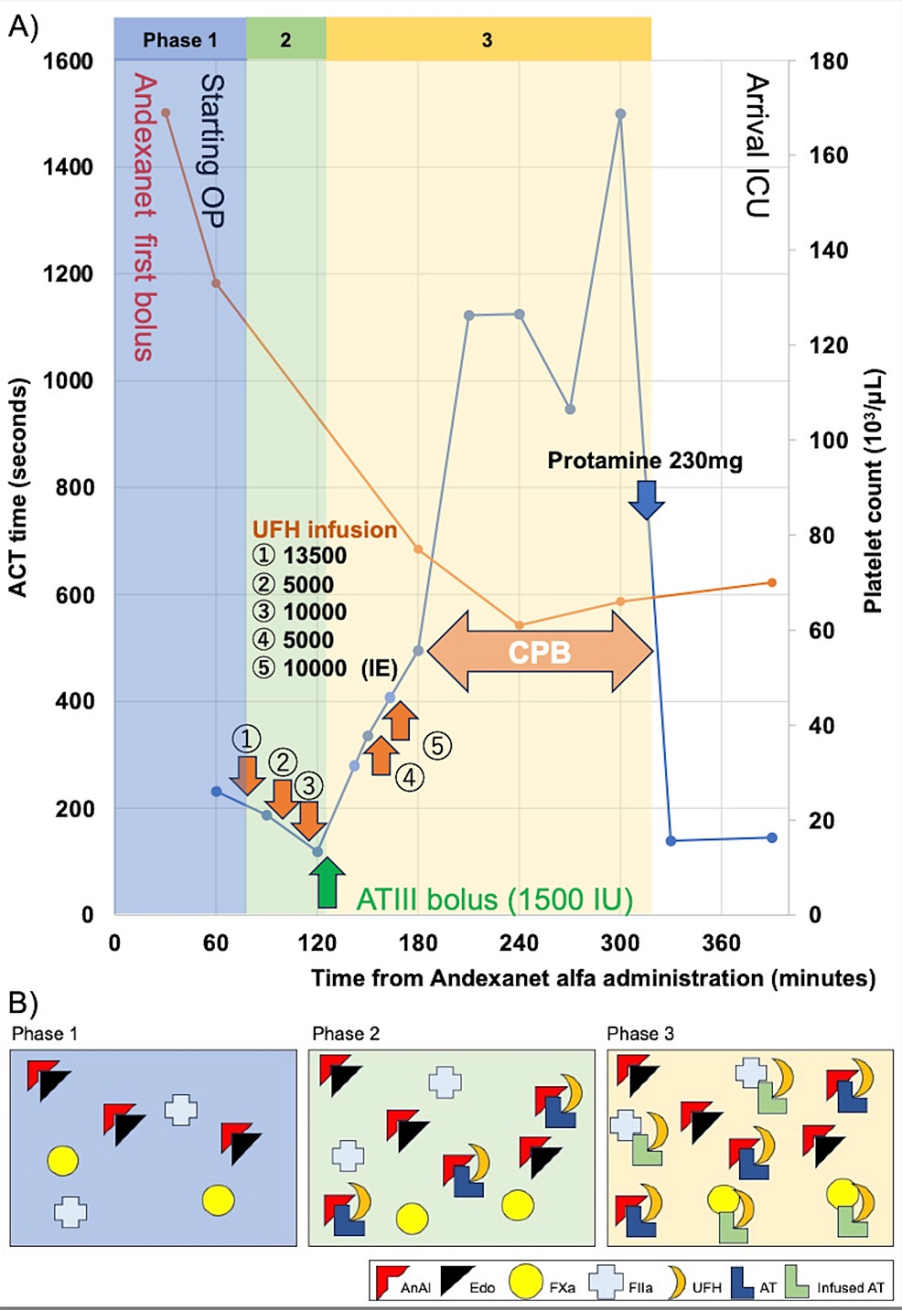
Timeline depicting ACT after AnAl administration and schematic representation of the potential mechanism for AnAl-induced heparin resistance. A) Timeline depicting ACT and platelet count during emergent aortic surgery. The ACT is on the left axis, and the platelet count is on the right axis. B) Schematic representation of the potential mechanism for AnAl-induced heparin resistance. In phase 1, AnAl combines with Edo. Phase 2 exhibits high binding affinity between AnAl and heparin-activated ATIII, leading to a reduction in the anticoagulant effect of heparin infusion. Phase 3 involves the infusion of ATIII, which combines with heparin-activated ATIII, resulting in an improvement in heparin resistance. ACT: activated clotting time; OP: operation; ICU: intensive care unit; UFH: unfractionated heparin; CPB: cardiopulmonary bypass; ATIII: antithrombin III; AnAl: andexanet alfa; Edo: edoxaban; FXa: factor Xa; FIIa: factor IIa; AT: antithrombin.

In this case, an 85-year-old woman weighing 45 kg presented with an acute type A aortic dissection (AAAD) complicated by cardiac tamponade. Her medical history included non-valvular atrial fibrillation managed with 30 mg of edoxaban and IgG4-related disease treated with 5 mg of prednisolone. She took her last dose of edoxaban on the morning of the emergent aortic repair. Upon admission, her blood pressure was 90 mmHg with a heart rate of 117 beats/min. Laboratory results showed liver dysfunction suspected to be due to cardiac tamponade, with serum aspartate aminotransferase at 521 U/L and alanine aminotransferase at 276 U/L. The preoperative values of the prothrombin time-international normalized ratio (PT-INR) were prolonged at 1.32, potentially due to DOACs or liver dysfunction. Activated partial thromboplastin time (APTT) and antithrombin III (ATIII) activity were at 27.6 seconds and 83%, respectively. The platelet count was 169,000/μL. A reversal therapy was initiated with a low dose of AnAl (400 mg bolus) as soon as possible. In accordance with the patient's and family's preferences, emergent surgical intervention was chosen to prevent further hemodynamic deterioration. The detailed surgical timeline after AnAl administration is depicted in Figure 1A.

The initial activated clotting time (ACT) measured before systemic heparinization was 232 seconds. Post-median sternotomy, despite an intravenous bolus of 13,500 IU (300 IU/Kg) of unfractionated heparin (UFH) was administered to achieve an ACT over 480 seconds, the ACT remained unaffected as follows: 187 seconds after the first dose (13,500 IU), 158 seconds after the seconds dose (5,000 IU), and 119 seconds after the third dose (10,000 IU) as shown in Figure 1A. Suspecting heparin resistance of some cause, 1,500 IU of ATIII (Takeda Pharmaceutical Company, Tokyo, Japan) was administrated, resulting in a significant impact on the extended ACT of 336 seconds. Alongside 15,000 IU of UFH, this had a substantial effect on the subsequent ACT, which reached 495 seconds. Subsequent measurements showed the APTT of 116.9 seconds and the PT-INR of 1.58.

Approximately 1,000 IU/kg of UFH was administered before establishing cardiopulmonary bypass (CPB). The ascending aortic repair with left atrial appendage closure was performed safely without blood clots on the heart-lung machine. We aimed for an ACT exceeding 480 seconds and successfully maintained over 980 seconds without additional UFH during CPB. The total CPB time, aortic cross-clamp time, and deep hypothermic arrest time were 146, 102, and 33 minutes, respectively. Adequate UFH reversal with 230 mg of protamine was confirmed through UFH concentration study using HMS plus (Medtronic, Inc., Minneapolis, Minnesota, USA). The measured ACT after protamine administration was 139 seconds, and good hemostasis was achieved in the surgical field. The blood loss during surgery amounted to 444 mL, with no bleeding complications during the procedure. At the time of admission to the intensive care unit, the ACT was 145 seconds, the APTT was 46.0 seconds, the PT-INR was 1.52, and the platelet count was 70,000/μL. 

## Discussion

This case report demonstrates that AnAl-induced heparin resistance can be effectively addressed through antithrombin administration, enabling the safe performance of emergent aortic surgery after reversal therapy with AnAl.

The requirement for high-dose UFH and the occurrence of blood clots in the CPB circuits due to AnAl-induced heparin resistance have been infrequently documented [1,5,6]. Al-Attar et al. reported a case of AAAD where pre-CPB AnAl administration necessitated the standard dose along with an additional 30,000 IU of UFH. However, the ACT did not exceed 290 seconds, and blood clots were observed in the CPB reservoir [1]. The preoperative use of AnAl and the resultant heparin resistance may pose potential risks during cardiac surgery requiring CPB, especially in patients who may require emergent CPB initiation. Furthermore, the duration of AnAl's effects is limited to up to 3 hours [1,4,5]. Therefore, we recommend avoiding AnAl administration before emergent cardiac surgery in the absence of immediate life-threatening bleeding and suggest early discontinuation of AnAl infusion if CPB is required during surgery.

The frequency of AnAl-induced heparin resistance, diagnostic methods, and appropriate treatment remain unknown. However, reports have indicated the risk of intraoperative blood clots associated with AnAl-induced heparin resistance during emergency cardiovascular surgery [1,5-10]. Therefore, in cases where AnAl has already been administered prior to systemic heparinization, a management protocol for AnAl-induced heparin resistance must be established. Although diagnostic criteria for heparin resistance have not been established, the use of thromboelastometry may be helpful to determine sufficient heparin dosage and to detect the presence of other coagulation abnormalities [6,9]. Supplementation of ATIII has shown to be a successful strategy for addressing this resistance [6,9]. The possible explanation for AnAl-induced heparin resistance lies in ATIII binding with AnAl, as depicted in Figure 1B. AnAl strongly interacts not only with DOACs but also with the heparin-activated ATIII complex. The reduction of heparin-activated ATIII complex in the blood, caused by AnAl binding, is believed to contribute to heparin resistance as shown in Figure 1B Phase 2 [6]. Thus, the infusion of ATIII supplementation could ameliorate this condition, as illustrated in Figure 1B Phase 3. In our case, the ACT was initially prolonged before heparin administration, potentially due to DOACs. However, the ACT subsequently shortened quite rapidly over time from AnAl administration despite the administration of heparin. The administration of a double routine dose of UFH still did not affect the ACT despite normal preoperative ATIII activity. However, a bolus of 1500 IU of ATIII had a significant impact on the ACT, and no thrombi were observed in the CPB circuits or surgical field during surgery.

Prior instances have reported thrombus formation resulting from inadequate ACT during the establishment of CPB [1,5,6]. Heparin resistance during endovascular repair for ruptured abdominal aortic aneurysms and thrombus formation due to performing procedures under insufficient ACT have been also documented [7,8]. Consequently, we strongly advocate initiating CPB only after the ACT exceeds an appropriate threshold to minimize the potential risk of clot formation within the CPB [1,5].

The impact of AnAl dosage on heparin resistance remains uncertain. Similarly, the ideal dosage of ATIII is not yet established. Apostel et al. noted an extension in ACT when administering 400 mg of AnAl followed by 1000 units of ATIII [6]. Honda et al. observed prolonged ACT with 3000 units of ATIII following an 880 mg dosage of AnAl [9]. In this case, ACT was extended using 1500 units of ATIII following a 400 mg dose. Conversely, Kitaura et al. reported that the administration of ATIII didn’t improve heparin resistance, while the administration of nafamostat mesylate was effective [10]. Moving forward, does heparin resistance vary with different AnAl doses? What is the optimal ATIII dose? Further case series are required to validate these inquiries.

## Conclusions

This case highlights the effective management of AnAl-induced heparin resistance during emergent aortic surgery through antithrombin administration. Concerns about high-dose UFH requirements and the risk of clot formation in CPB circuits due to AnAl-induced heparin resistance have been sporadically documented in the literature. Our case aligns with these concerns, emphasizing the challenges associated with AnAl administration precluding adequate ACT attainment and posing risks of intraoperative clotting in CPB. To mitigate these risks, we recommend avoiding AnAl administration before emergent cardiac surgery unless in cases of immediate life-threatening bleeding. Furthermore, prompt discontinuation of AnAl infusion before CPB initiation is advised. AnAl exhibits adequate hemostatic efficacy when administered after heparin reversal. If AnAl has been administered before systemic heparinization, establishing a protocol for managing AnAl-induced heparin resistance becomes crucial. ATIII supplementation serves as a successful strategy to counteract this resistance, possibly by addressing the reduction of heparin-activated ATIII complex caused by AnAl binding. Our case emphasizes the importance of careful planning, ATIII supplementation, and appropriate ACT monitoring to minimize the risk of intraoperative clot formation in CPB circuits during emergent cardiac surgery following AnAl administration. Clinicians involved in cardiac surgery should be cognizant of the potential risk of heparin resistance associated with AnAl. Further research is warranted to establish the optimal timing of AnAl infusion and the ideal dosage of ATIII to manage AnAl-induced heparin resistance in cardiac surgery.
